# Prospective Activity of PLG0206, an Engineered Antimicrobial Peptide, on Chronic Periprosthetic Joint Infection Total Knee Arthroplasty Components *Ex Vivo*: The Knee Explant Analysis (KnEA) Study

**DOI:** 10.1128/Spectrum.01879-21

**Published:** 2021-11-24

**Authors:** David Huang, Dana M. Parker, Jonathan B. Mandell, Kimberly M. Brothers, Charles G. Gish, John A. Koch, Nicholas Pachuda, Despina Dobbins, Jonathan Steckbeck, Kenneth L. Urish

**Affiliations:** a Peptilogics, Pittsburgh, Pennsylvania, USA; b Arthritis and Arthroplasty Design Group, Department of Orthopedic Surgery, University of Pittsburgh, Pittsburgh, Pennsylvania, USA; c The Bone and Joint Center, Magee-Womens Hospital of the University of Pittsburgh Medical Center, Pittsburgh, Pennsylvania, USA; d Department of Bioengineering, University of Pittsburgh, Pittsburgh, Pennsylvania, USA; e Clinical and Translational Science Institute, University of Pittsburgh, Pittsburgh, Pennsylvania, USA; National University Hospital

**Keywords:** PJI, PLG0206, biofilms

## Abstract

PLG0206 is an engineered antimicrobial peptide that has completed phase 1 clinical studies. A prospective study was completed on explanted implants from chronic periprosthetic joint infections (*n* = 17). At a concentration of 1 mg/mL for 15 min, there was a mean 4-log_10_ reduction (range, 1 to 7) in the bacterial CFU identified from the implants.

**IMPORTANCE** Chronically infected prosthetics of the knee were exposed to PLG0206, an engineered antimicrobial peptide, at a concentration of 1 mg/mL for 15 min. A mean 4-log_10_ reduction (range, 1 to 7) in the number of bacteria occurred, which may translate to improved clinical outcomes for persons with prosthetic joint infection of the knee.

## OBSERVATION

Periprosthetic joint infection (PJI) is the leading cause of revision total knee arthroplasty, with high morbidity and mortality. The incidence of PJI is 1 to 3% in primary arthroplasty (1) and 3 to 10% in revision arthroplasty (2, 3). The incidence of PJI is projected to increase to 10,000 cases per year by 2030 (2). Debridement, antibiotics, and implant retention (DAIR) is a conservative treatment option for acute perioperative infection or an acute hematogenous infection of the knee. Unfortunately, this treatment is associated with failure rates of approximately 60% (4–10).

PLG0206 is a rationally designed, engineered antimicrobial peptide that is broad spectrum, rapidly acting, and active against antibiotic-tolerant biofilm (11, 12). The purpose of this study was to prospectively evaluate the *ex vivo* activity of PLG0206 at an expected clinical concentration of at least 1 mg/mL for 15 min on explanted components from total knee arthroplasty (TKA) PJI. The primary objective was to determine the reduction in bacterial count compared to that in untreated explants.

From 25 January 2021 to 5 August 2021, 17 adult patients presented with chronic bacterial total knee arthroplasty (TKA) PJI at the University of Pittsburgh Medical Center Healthcare System (UPMC); despite receiving chronic suppressive oral/intravenous antibiotics, they required a 2-stage revision procedure for explantation of components. All patients met the criteria for a diagnosis of PJI as defined by the 2018 International Consensus Meeting (13). The infected prosthetics were removed and deidentified. PLG0206 was diluted in phosphate-buffered solution (PBS) at a concentration of 1 mg/mL and adjusted to pH 7.40. The removed implant prosthetics were submerged *ex vivo* with PLG0206 at an expected clinical concentration of 1 mg/mL for 15 min. The PLG0206 concentration of 1 mg/mL for 15 min was selected based off a series of *in vitro* time-kill studies and previous murine and rabbit PJI animal models (2-log reduction of Staphylococcus aureus CFU isolated from implant material following treatment with PLG0206 in combination with a DAIR procedure) (11). The explants were first rinsed with 50 mL PBS and then treated with 1 mg/mL PLG0206. After a 15-minute exposure to PLG0206, the explants were rinsed with 50 mL PBS, placed in PBS containing 1% Tween 20 (PBST), then sonicated for 10 min to disrupt the biofilm on the explant surface (14). Previous *in vitro* studies using the same sonication protocol did not demonstrate significant improvements in PLG0206 activity (11). The sonicated solution was then serially diluted and plated onto Trypticase soy agar (TSA) II sheep blood agar plates to determine the antibiotic sensitivity and bacterial burden determination in CFU per milliliter. The remaining explanted implant material from the same patient was sonicated and served as an untreated control. Quantitative culture was directly performed on the untreated sonicate, and when this was not possible, the CFU were estimated from the reported clinical value in the medical record. If a sample was deemed “too numerous to count,” the CFU were determined by serially diluting the sonicate. Fourteen of seventeen (82.4%) patients received antibiotics prior to the 2-stage revision procedure (Table 1). Both Gram-positive and Gram-negative bacteria were identified from the removed prosthetics during the 2-stage revision procedure for chronic bacterial PJI. The most common bacteria identified from the prosthesis were Staphylococcus epidermidis (6/17; 35%), Staphylococcus aureus (3/17; 18%), and Escherichia coli (2/17; 12%). The majority (11/17; 65%) of the bacteria were resistant to at least one antibiotic (Table 1). Ten out of seventeen samples (59%) of the chronically infected prosthetics treated *ex vivo* with 1 mg/mL PLG0206 became culture negative. The infected prosthetics exposed to PLG0206 demonstrated a mean 4-log_10_ reduction (range, 1 to 7), whereas those not exposed to PLG0206 did not demonstrate any reduction in the bacterial burden. There were 7 samples, primarily at the beginning of the study, where estimates of the untreated bacterial burden were used because quantitative cultures were not available. All but one of these samples were culture negative after treatment with PLG0206. If these estimates are excluded from analysis, there was a mean 2.5-log_10_ reduction (range, 1 to 4).

**TABLE 1:** Culture and CFU log reduction among bacteria identified from periprosthetic knee joints exposed and not exposed to PLG0206^*a*^

Prosthetic no.	Preoperative antibiotics?	Culture	Resistance pattern	CFU/mL untreated	CFU/mL treated
1	Yes (cephalexin)	Staphylococcus epidermidis	Clindamycin, erythromycin, gentamicin, oxacillin	5 × 10^7^^*b*^	Culture negative
2	Yes (cephalexin)	Staphylococcus epidermidis	Clindamycin, erythromycin, gentamicin, oxacillin	5 × 10^7^^*b*^	Culture negative
3	No	Staphylococcus aureus (MRSA)	Oxacillin, erythromycin	5 × 10^7^^*b*^	Culture negative
4	Yes	Staphylococcus haemolyticus	Clindamycin, gentamicin, oxacillin, rifampin, TMP/SMX	7.3 × 10^2^	Culture negative
5	Yes (TMP/SMX)	Staphylococcus aureus (MSSA)	Susceptible	5 × 10^7^^*b*^	12.5 × 10^3^
6	Yes	Staphylococcus caprae	Susceptible	5 × 10^7^^*b*^	Culture negative
7	Yes (cefuroxime)	Escherichia coli	Ampicillin, ampicillin/sulbactam	3.5 × 10^4^	6 × 10^1^
8	Yes (cefuroxime)	Escherichia coli	Ampicillin, ampicillin/sulbactam	3.5 × 10^4^	3 × 10^1^
9	No	Staphylococcus epidermidis	Susceptible	1.9 × 10^5^	9 × 10^1^
10	Yes (doxycycline)	Haemophilus parainfluenzae	Susceptible	5 × 10^7^^*b*^	Culture negative
11	Yes (doxycycline)	Haemophilus parainfluenzae	Susceptible	5 × 10^7^^*b*^	Culture negative
12	Yes (ciprofloxacin)	Enterococcus faecalis	Susceptible	1.3 × 10^5^	1 × 10^1^
13	Yes (vancomycin)	Staphylococcus aureus (MRSA)	Oxacillin, erythromycin	1.1 × 10^5^	Culture negative
14	Yes (vancomycin and cefepime)	Streptococcus dysgalactiae	Susceptible	6 × 10^1^	Culture negative
15	No	Staphylococcus epidermidis	Penicillin	3.2 × 10^5^	Culture negative
16	Yes (cephalexin)	Staphylococcus epidermidis	Oxacillin, tetracycline, TMP/SMX	3.2 × 10^3^	Culture negative
17	Yes (cephalexin)	Staphylococcus epidermidis	Oxacillin, tetracycline, TMP/SMX	3.2 × 10^3^	1 × 10^1^

a MRSA, methicillin-resistant S. aureus; MSSA, methicillin-sensitive S. aureus; TMP/SMX, trimethoprim-sulfamethoxazole.

b Estimate of the CFU in untreated samples.

The microorganisms identified from the implant prosthetics are consistent with previously published studies (15, 16), in which S. aureus and coagulase-negative staphylococci (CoNS) contribute to between 50 and 60% of PJIs. CoNS species, of which S. epidermidis was the most frequently identified pathogen of this group, are ubiquitous members of the human microbiome found on the skin. The relative pathogenicity of these microorganisms is unclear. However, both S. aureus and CoNS cause PJI primarily through their ability to adhere to prosthetic materials, produce biofilm, and produce virulence factors. In most studies, the most commonly isolated aerobic Gram-negative bacillus is E. coli (15, 16).

Many of the bacteria identified from the infected prosthetics were susceptible to the antibiotic the patient was prescribed prior to prosthetic removal. Biofilm formation may have protected the bacteria from the antibiotics as well as the host immune system, making treatment of the infection difficult without a biofilm-directed treatment strategy. Given the limitations of treatments currently available, this mandates surgical intervention, in many cases including complete removal of the prosthesis, in order to achieve infection control. The limited susceptibility of bacteria in biofilm is related to their low growth rate, the presence of resistant bacterial subpopulations, and a microenvironment within the biofilm that impairs antimicrobial activity (17–19). Biofilm formation may also explain why some normal floral organisms traditionally considered “harmless” (e.g., coagulase-negative staphylococci) become pathogens when they are grown in the presence of foreign bodies.

In general, antimicrobial therapy should be pathogen directed and guided by the results of antimicrobial susceptibility testing, where applicable. However, most antimicrobials do not have antibiofilm activity. PLG0206 has broad-spectrum activity, including activity against multidrug-resistant bacteria that cause PJI, has potent activity against antibiotic-resistant biofilm, does not have significant local or systemic toxicity in the therapeutic range of dosing in animal models, and has pharmacokinetics with a half-life of more than 12 h (11, 12). In this study, after 15 min of exposure to an expected clinical concentration of 1 mg/mL, a mean 4-log_10_ reduction in CFU counts was observed among the prosthetics exposed to PLG0206 in comparison to those that were not. These findings support the development of PLG0206 as a local irrigation solution of at least a 1 mg/mL concentration in the wound cavity for 15 min for patients undergoing treatment of a PJI occurring after TKA or total hip arthroplasty (THA).
